# A *KHDC3L* mutation resulting in recurrent hydatidiform mole causes genome-wide DNA methylation loss in oocytes and persistent imprinting defects post-fertilisation

**DOI:** 10.1186/s13073-019-0694-y

**Published:** 2019-12-17

**Authors:** Hannah Demond, Zahra Anvar, Bahia Namavar Jahromi, Angela Sparago, Ankit Verma, Maryam Davari, Luciano Calzari, Silvia Russo, Mojgan Akbarzadeh Jahromi, David Monk, Simon Andrews, Andrea Riccio, Gavin Kelsey

**Affiliations:** 1 Epigenetics Programme, Babraham Institute, Cambridge, UK; 2 Infertility Research Center, Shiraz University of Medical Sciences, Shiraz, Iran; 3 Department of Obstetrics and Gynecology, School of Medicine, Shiraz University of Medical Sciences, Shiraz, Iran; 4 Institute of Genetics and Biophysics ‘Adriano Buzzati-Traverso’, Consiglio Nazionale delle Ricerche (CNR), Naples, Italy; 5 Dipartimento di Scienze e Tecnologie Ambientali Biologiche e Farmaceutiche, Università degli Studi della Campania ‘Luigi Vanvitelli’, Caserta, Italy; 6 IVF Section, Ghadir-Mother and Child Hospital of Shiraz, Shiraz, Iran; 7 Medical Cytogenetics and Molecular Genetics Laboratory, Centro di Ricerche e Tecnologie Biomediche IRCCS, Istituto Auxologico Italiano, Milan, Italy; 8 Department of Pathology, Shiraz University of Medical Sciences, Shiraz, Iran; 9 Imprinting and Cancer Group, Bellvitge Biomedical Research Institute (IDIBELL), L’Hospitalet de Llobregat, Barcelona, Spain; 10 Bioinformatics Group, Babraham Institute, Cambridge, UK; 11 Centre for Trophoblast Research, University of Cambridge, Cambridge, UK

**Keywords:** Epigenetics, DNA methylation, Genomic imprinting, Single-cell analysis, Oocytes, Embryos

## Abstract

**Background:**

Maternal effect mutations in the components of the subcortical maternal complex (SCMC) of the human oocyte can cause early embryonic failure, gestational abnormalities and recurrent pregnancy loss. Enigmatically, they are also associated with DNA methylation abnormalities at imprinted genes in conceptuses: in the devastating gestational abnormality biparental complete hydatidiform mole (BiCHM) or in multi-locus imprinting disease (MLID). However, the developmental timing, genomic extent and mechanistic basis of these imprinting defects are unknown. The rarity of these disorders and the possibility that methylation defects originate in oocytes have made these questions very challenging to address.

**Methods:**

Single-cell bisulphite sequencing (scBS-seq) was used to assess methylation in oocytes from a patient with BiCHM identified to be homozygous for an inactivating mutation in the human SCMC component KHDC3L. Genome-wide methylation analysis of a preimplantation embryo and molar tissue from the same patient was also performed.

**Results:**

High-coverage scBS-seq libraries were obtained from five *KHDC3L*^c.1A>G^ oocytes, which revealed a genome-wide deficit of DNA methylation compared with normal human oocytes. Importantly, germline differentially methylated regions (gDMRs) of imprinted genes were affected similarly to other sequence features that normally become methylated in oocytes, indicating no selectivity towards imprinted genes. A range of methylation losses was observed across genomic features, including gDMRs, indicating variable sensitivity to defects in the SCMC. Genome-wide analysis of a pre-implantation embryo and molar tissue from the same patient showed that following fertilisation methylation defects at imprinted genes persist, while most non-imprinted regions of the genome recover near-normal methylation post-implantation.

**Conclusions:**

We show for the first time that the integrity of the SCMC is essential for de novo methylation in the female germline. These findings have important implications for understanding the role of the SCMC in DNA methylation and for the origin of imprinting defects, for counselling affected families, and will help inform future therapeutic approaches.

## Background

The subcortical maternal complex (SCMC), a multi-protein structure of the mammalian oocyte, orchestrates a number of essential cellular processes during the oocyte-to-embryo transition, such as spindle assembly, chromosome alignment and symmetric cell division in cleavage-stage embryos [1, 2]. In humans, mutations in SCMC proteins cause various developmental abnormalities, including early embryonic arrest and reproductive failure [3–9]. A recurrent, but unexplained, finding is abnormalities in genomic imprinting. Maternal recessive mutations in *NLRP7* and *KHDC3L*, both indicated to encode SCMC components [3, 4, 10, 11], are the predominant cause of biparental, complete hydatidiform mole (BiCHM; also referred to as recurrent, familial hydatidiform mole; OMIM 231090 and 614293), a rare gestational abnormality characterised by trophoblast overgrowth and absence of embryo development. *NLRP7* or *KHDC3L* mutations are found in the majority of BiCHM cases (*NLRP7* ~ 75%, *KHDC3L* 5–10%) and are associated with widespread loss of methylation (LoM) of germline differentially methylated regions (gDMRs) of imprinted genes in molar tissue [3, 4, 12, 13]. In addition, mutations in other SCMC components, including PADI6, OOEP, NLRP5 and NLRP2, have been described in single imprinting syndromes or multi-locus imprinting disturbance (MLID), and PADI6 has been associated with molar pregnancies [5, 8, 14]. However, the molecular aetiology of BiCHM and MLID is obscure, as connections between the SCMC under the oocyte plasma membrane and the nuclear DNA methylation machinery have not been defined. This lack of molecular understanding prevents a meaningful development of therapeutic approaches or satisfactory counselling of affected families. Mouse models have thus far not been informative, because there are no direct homologues of *KHDC3L* or *NLRP7*, and because maternal effect mutations in *Nlrp5/Mater*, *Ooep/Floped* and *Tle6* lead to very early developmental arrest [1, 15, 16]. Analysis of a mouse *Nlrp2* knockout, which is compatible with development to term but with reduced fertility, has implicated a defect in methylation maintenance of imprinted genes post-fertilisation. It was shown that localisation of the maintenance DNA methyltransferase DNMT1 with the SCMC is disrupted in *Nlrp2* knockout oocytes while DNMT3A, the predominant methyltransferase responsible for de novo methylation in the oocyte, retains normal chromosome association [17]. However, while mid-gestation embryos and neonates from *Nlrp2*-deficient oocytes exhibit limited methylation alterations of some imprinted genes, there is no generalised LoM of imprints typical of molar tissue [17]. Furthermore, with the rarity of BiCHM, especially with a *KHDC3L* mutation, and because patients do not benefit from assisted reproduction techniques other than egg donation, human oocyte or early embryo material to study the origins of BiCHM and MLID has been even more difficult to obtain. Therefore, until now, there has been no assessment of whether DNA methylation defects occur in oocytes either in mouse models or human cases. Amongst the key outstanding questions are (1) whether there is a defective establishment of methylation in oocytes or a failure to maintain methylation specifically at imprinted loci in pre-implantation embryos during genome-wide erasure of gametic methylation and (2) whether LoM is limited to imprinted loci or is more widespread. All these possibilities could pertain because, in addition to undergoing genome-wide de novo methylation [18], the oocyte provides the key factors required for the complex methylation reprogramming events in the cleavage-stage embryo, including gDMR methylation maintenance [19, 20]. Genome-wide methylation analysis of BiCHM cases with *NLRP7* mutations using methylation arrays reveals a widespread and apparently selective effect on imprinted gDMRs [13], but this may not reflect the methylation defect as it originates in the oocyte or pre-implantation embryo, because molar tissues were examined after remethylation of the genome at implantation. Until the development of single-cell genome-wide methylation profiling (scBS-seq) [21], it has been impossible to answer these long-standing questions. Here, we had the unique opportunity to examine methylation both in oocytes and molar tissue from the same patient homozygous for a loss-of-function mutation in *KHDC3L*. We found that DNA methylation establishment in the oocyte was globally impaired and that this LoM persisted at imprinted loci until post-implantation. This is the first study to assess the origin of an imprinting disorder in the human germline, demonstrating the importance of the integrity of the SCMC for DNA methylation establishment in the oocyte.

## Methods

### Patient details

Patient D was registered at the Mole Clinic of the Shiraz University of Medical Sciences with a history of two successive complete hydatidiform molar pregnancies and identified by the Infertility Research Centre for subsequent mutation testing. A *KHDC3L* mutation was confirmed using Sanger sequencing.

### Tissue sample preparation

A tissue sample from one of the molar conceptuses and an endometrium tissue sample from patient D were collected and formalin-fixed paraffin-embedded (FFPE) for further analysis. As controls, FFPE tissue samples were obtained from the placenta from two control patients, as well as maternal endometrium and molar tissue from a patient with a sporadic case of androgenic complete hydatidiform mole (AnCHM). DNA was extracted using the QIAamp DNA FFPE Tissue Kit (Qiagen), and contamination of molar tissue was tested by Chromoquant QF-PCR kit (CyberGene AB).

### Oocyte collection and ICSI procedure

Oocytes were obtained voluntarily from patient D at the IVF centre of the Ghadir Mother and Child Hospital affiliated to Shiraz University of Medical Sciences with signed informed consent of the patient and her husband and the approval of the Ethics Committee of Shiraz University of Medical Sciences (ethics codes: IR.sums.rec.1395.S718 for oocyte retrieval and IR.sums.rec.1396.S779 for embryo production). Mature oocytes were obtained after ovarian stimulation using a standard gonadotropin-releasing hormone (GnRH) antagonist protocol. Oocytes were collected in G-IVF plus (Vitrolife) and cleaned in G-MOPS (Vitrolife) supplemented with 80 IU/ml hyaluronidase (HYASE-10X, Vitrolife). Out of nine oocytes, seven were collected for subsequent scBS-seq analysis. Intracytoplasmic sperm injection (ICSI) was performed followed by 6 days embryo culture with the two remaining oocytes, resulting in one embryo, which was collected in < 5 μl RLT buffer for whole-embryo BS-seq analysis.

### DNA methylation array

DNA methylation of FFPE tissue samples was analysed by chip array. DNA was sodium bisulphite treated using the EZ DNA Methylation Kit (D5001, Zymo Research). Single-strand bisulphite-converted DNA was quantified with the NanoPhotometer Pearl (Implen GmbH), and DNA length was restored using the Infinium HD FFPE Restore Kit (WG-321-1002, Illumina). Genome-wide methylation was performed on the Infinium MethylationEPIC Bead Chip (WG-317-1001, Illumina) following Infinium HD FFPE Methylation Assay instructions and using Illumina-supplied reagents and conditions. Fluorescence intensities were captured using Illumina HiScan SQ (Illumina). The methylation profiles of 2 of the 11 control placentas were processed together with the samples of the *KHDC3L*^c.1A>G^ and AnCHM patient. The other 9 control placentas were processed separately.

### DNA methylation array analysis

The chip array data were analysed using R (v. 3.5.3). Beta values were extracted from ‘idat’ files by using the ‘Load’ module of the ‘Champ’ R package (v. 2.12.0) [22], with quality control options set as default. After this quality control step, 745,259 probes were retained and used for further analysis. SWAN normalisation [23] was applied, with the ‘method’ option set to ‘minfi’. The SWAN-normalised samples were assigned with respective genome coordinates based on probe name and manifest file (Illumina). The coordinates for the array data were then converted to human genome version hg38 (GRCh38) using CrossMap (version 0.2.5) [24].

A genome-wide analysis was performed with a tile-based approach using the ‘makewindows’ parameter of bedtools (v2.25.0) [25] to generate fixed-sized 20-kb consecutive genome windows to compare across samples (*n* = 160,724) of which 101,127 were retained after the intersection with the array data. Further filtering was applied to ensure a minimum of 3 informative CpGs covered by the array per 20-kb window. Only windows with the required minimal coverage in all samples were taken into account, resulting in the assessment of 65,339 windows (64.6%).

To analyse DNA methylation of imprinted DMRs, annotations of gDMRs were taken from Sanchez-Delgado et al. [13] and Hanna et al. [26] and categorised as described in Additional file 1: Table S1. The coordinates of the array data were intersected with DMRs viz. classic maternal and paternal gDMRs, placenta-specific gDMRs and secondary DMRs using bedtools. Probes over CpG islands (CGIs) were defined using CpG island features from EnsEMBL v90. Placenta methylated (> 70%) and unmethylated (< 20%) CGIs were filtered using publically available whole-genome bisulphite sequencing (WGBS) DNA methylation data of placenta [27] as a reference. Non-CGI regions were called by filtering 20-kb windows that were not overlapping CGIs. To analyse the DNA methylation loss in different genomic regions, genome features from EnsEMBL v90 were used. Plots were generated using the R packages ggplot2 for the scatter plot and PCA, gplots (heatmap.2 function) for heatmaps and ggpubr R package for boxplots. The methylation profile in array data was visualised using the UCSC genome browser (GRCh38). Hypomethylated regions in the *KHDC3L*^c.1A>G^ mole were defined as 20-kb windows with a mole/control placenta methylation ratio of < 0.65 and at least ten covered CpGs per window.

### Pyrosequencing

DNA from FFPE tissues was bisulphite converted using the EpiTect Bisulfite Kit (Qiagen). Methylation analysis (CpG) pyrosequencing assays were designed with PyroMark Assay Design SW 2.0. Primers sequences are listed in Additional file 2: Table S2. The PyroMark PCR kit (Qiagen) was used to amplify 200 ng of converted DNA. Quantitative DNA methylation analysis was performed using the PyroMark Q48 Autoprep, according to the manufacturer’s instructions (Qiagen). The results were analysed with the Q-CpG software (V.1.0.9Pyrosequencing).

### Bisulphite sequencing

DNA methylation of single oocytes was assessed using WGBS according to the single-cell adaptation (scBS-seq) of the post-bisulphite adaptor tagging (PBAT) method as previously described [21, 28]. The PBAT protocol was also employed to analyse DNA methylation of the embryo, using a slightly adapted method for bulk samples as described elsewhere [29]. Single-cell libraries were amplified for 14 cycles, and the embryo library was amplified for 12 cycles. Libraries were sequenced on the Illumina MiSeq to assess library quality and to screen for somatic cell contamination. Good-quality libraries were then sequenced deeper on the Illumina NextSeq platform.

### Public datasets

As controls for our DNA methylation data, we used publically available datasets. Raw sequencing reads were obtained from DDBJ and GEO databases (https://www.ddbj.nig.ac.jp/index-e.html and https://www.ncbi.nlm.nih.gov/geo/) for the following datasets and processed with the parameters detailed below: bulk DNA methylation from oocytes, sperm and blastocysts (accession DRP002710) [30]; single-cell DNA methylation from oocytes and embryos (accession GSE81233) [31]; bulk DNA methylation from the placenta (accession GSM1186665) [27]; and single-cell RNA sequencing from oocytes (accession GSE44183) [32].

### Library mapping and trimming

Raw fastq sequence files were initially quality trimmed and adaptor trimmed with Trim Galore v0.4.2. Single-cell data were trimmed with the ‘--clip_r1 9’ parameter added in a single-end mode. Bulk PBAT data was trimmed with the ‘--clip_r1 9’. RNA-seq data were clipped with default parameters. Mapping and methylation calling of bisulphite sequencing data were performed with Bismark v0.19.1 against the human GRCh38 genome assembly. PBAT data were called using the --pbat mode. Single-cell bisulphite data were called using the --non_directional mode. RNA-seq data were mapped against the human GRCh38 genome assembly using Hisat2 v2.1.0 using --no-softclipping and --dta and guided by splice junctions extracted from EnsEMBL v94.

### DNA methylation sequencing analysis

DNA methylation was quantified in the *KHDC3L*^c.1A>G^ oocytes and control oocytes in SeqMonk. Individual *KHDC3L*^c.1A>G^ oocyte libraries were compared to published single-cell libraries [31]. Since DNA methylation in the *KHDC3L*^c.1A>G^ oocytes appeared similar, datasets were grouped for part of the analysis to increase genome-wide coverage of CpGs and compared to grouped single-cell datasets of MII oocytes [31] and a bulk dataset of GV/MI oocytes [30]. Using Seqmonk, a tile-based method was applied to bin consecutive genomic windows with a fixed length (50 kb for single-cell and 20 kb for grouped analysis) to facilitate comparison across individual samples. Methylation values were quantified with bisulphite sequencing pipeline quantification, which calculates per-base methylation percentages and then averages these within each window. Filters were applied to ensure a minimum coverage of five or ten observed cytosines per probe window for single-cell and grouped data analysis, respectively. Only windows with the required minimal coverage in all samples were taken into account, allowing for the assessment of 26.6% of probes (*n* = 16,448) for single-cell analysis and 92.1% of probes (*n* = 142,204) for grouped data analysis. The initial quality of single-cell oocyte libraries was analysed by MiSeq using 100-kb overlapping windows with a 10-kb step size. Methylated and unmethylated domains were called using publically available DNA methylation data in oocytes and sperm [30] as a reference. Adjacent 10-kb windows with > 70% or < 30% DNA methylation, respectively, were merged and filtered for a size of ≥ 50 kb.

DNA methylation maintenance was analysed by calculating the embryo to oocyte methylation ratio, under the assumption that perfect maintenance would be reflected as a ratio of 0.5. Only the regions that had ≥ 20% residual methylation in *KHDC3L*^c.1A>G^ oocytes were included in the maintenance analysis. Methylated domains were filtered for those that were uniquely methylated in one parental germline but not the other. Probes over CGIs were defined using CpG island features from EnsEMBL v90. Again, DNA methylation of control oocytes was used to filter for methylated CGIs (> 70%) and unmethylated CGIs (< 20%). Annotations of gDMRs are listed in Additional file 1: Table S1. Genome features used to assess DNA methylation loss were from EnsEMBL v90. For analysis of repetitive elements, sequencing libraries were mapped against of prototypic consensus sequences of repetitive DNA elements, extracted from Repbase in 2015 (https://www.girinst.org/repbase/). Average DNA methylation for each element was quantitated. Repetitive elements were grouped into classes (ERV, L1, LTR, SINE, microsatellite repeats). Microsatellite repeats were excluded from further analysis because of insufficient coverage. CpGs in ZFP57-binding sites were defined as CpGs within the hexanucleotide TGCCGC described by Quenneville et al. [33] and alternative binding site GGCCGC from Anvar et al. [34] that were contained within maternal gDMRs (390 binding sites). The percentage of methylated binding sites was calculated for each single cell (control and *KHDC3L*^c.1A>G^).

### Statistical analysis

Statistical analysis was conducted using GrapPad Prism 7 and R. Normal distribution of datasets was analysed using a Shapiro-Wilk normality test. DNA methylation differences between *KHDC3L*^c.1A>G^ mole and control placenta were analysed with a Wilcoxon signed-rank test. Differences between *KHDC3L*^c.1A>G^ and control oocytes in global CpG methylation and methylation of ZFP57-binding sites were determined using an unpaired two-tailed *t* test. The Wilcoxon signed-rank test was employed to assess DNA methylation differences between *KHDC3L*^c.1A>G^ and control oocytes of methylated and unmethylated domains, methylated and unmethylated CGIs, and maternal gDMRs, as well as the DNA methylation maintenance ratio. To determine the effect size of DNA methylation changes in gDMRs, methylated CGIs, unmethylated CGIs and non-CGI regions in *KHDC3L*^c.1A>G^ mole and control placenta, a Brown-Forsythe and Welsh ANOVA followed by Dunnett’s T3 multiple comparisons test were carried out.

## Results

### Methylation defects in biparental complete hydatidiform mole caused by *KHDC3L* mutation

**Fig. 1:**
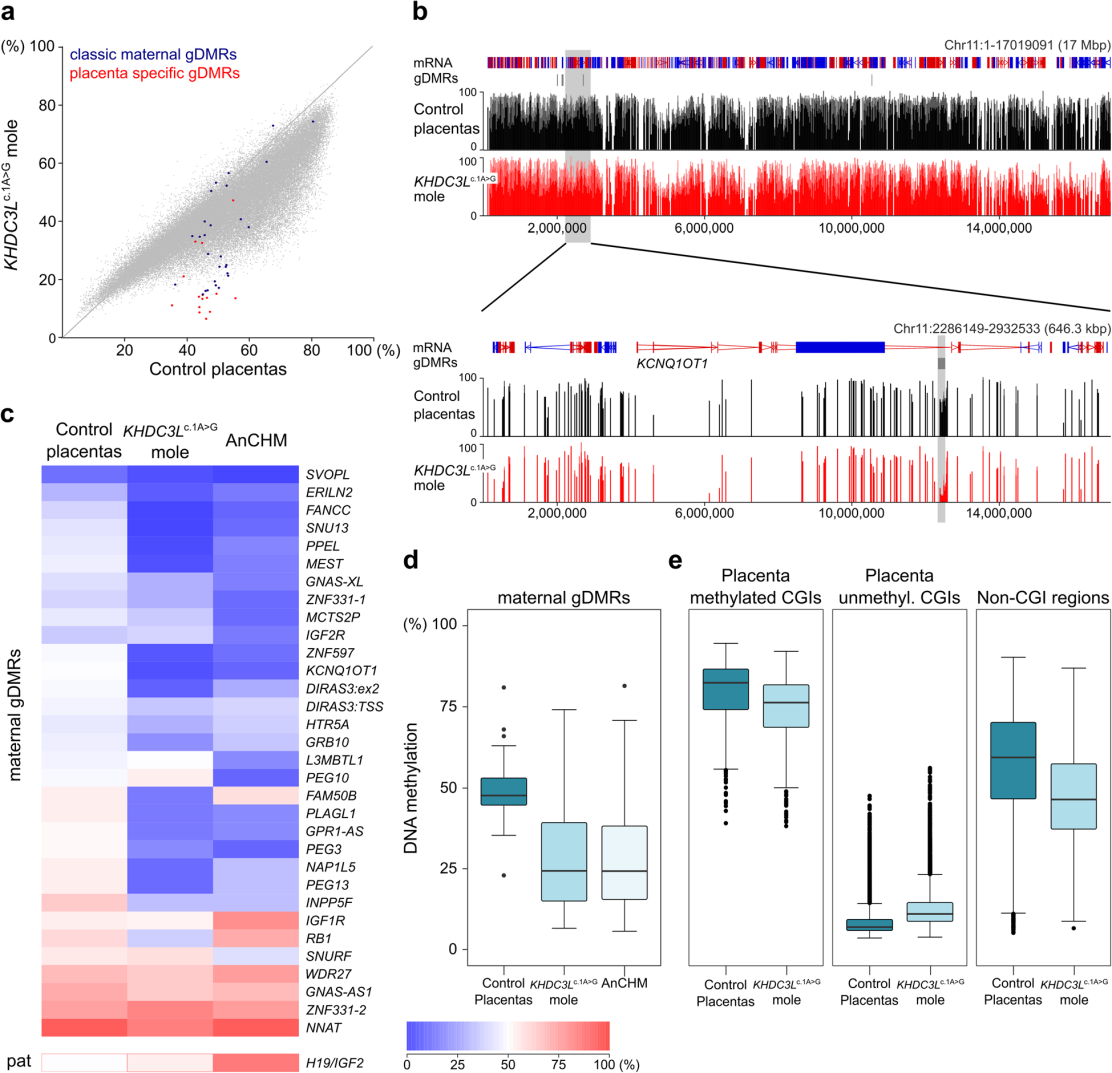
DNA methylation analysis of molar tissue of a patient with a *KHDC3L* mutation. **a** Scatterplot of methylation values from the Illumina Human MethylationEPIC BeadChip array of 20-kb windows in the *KHDC3L*^c.1A>G^ mole compared with the grouped control placentas (*n* = 11). Highlighted are methylation levels of classic and placenta-specific maternal gDMRs. **b** UCSC browser screenshot of genomic methylation in *KHDC3L*^c.1A>G^ mole compared with the control placentas (*n* = 11). The zoomed-in view shows methylation over the *KCNQ1OT1* imprinted domain with gDMR location highlighted in grey. Each vertical bar represents a single CpG on the array. **c** Heatmap showing methylation of maternal and paternal gDMRs in the *KHDC3L*^c.1A>G^ mole, control placentas (*n* = 11) and AnCHM. **d**, **e** Box and whisker plots showing methylation in the control placentas (*n* = 11) and *KHDC3L*^c.1A>G^ molar tissue at **d** maternal gDMRs (*n* = 43) and **e** placenta-specific methylated CGIs (*n* = 937), placenta-specific unmethylated CGIs (*n* = 11,728) and non-CGI windows (*n* = 48,581). Boxes represent the interquartile range; lines, the median; and dots beyond the whiskers, outliers. Comparisons in **d** and **e** are all statistically significant (*p* < 0.0001), although the effect size at maternal gDMRs is significantly greater (Additional file 3: Fig. S3E). Detailed statistical measures are given in Additional file 4: Table S3

Patient D, a 27-year-old woman homozygous for a confirmed loss-of-function mutation (A to G at the + 1 position in start codon) in *KHDC3L* with multiple consanguineous marriages in her extended family (Additional file 3: Figure S1A,B), had a history of 2 BiCHMs. A sample of 1 of these molar conceptuses with no evidence of contamination from maternal tissue (Additional file 3: Figure S2A) was investigated. The presence of 2 alleles with balanced peaks for each informative microsatellite marker analysed confirmed that the molar tissue was derived from a biparental conceptus (Additional file 3: Figure S2A). Similar to patient D, the husband and the *KHDC3L*^c.1A>G^ mole were found to be homozygous for the *KHDC3L* mutation (Additional file 3: Figure S1A and S2B); this result, which could be explained by the parents’ consanguinity (Additional file 3: Figure S1B), excludes the presence of normal KHDC3L in the conceptus as well as in oocytes. We used the Illumina Human MethylationEPIC BeadChip array to assess the genome-wide DNA methylation profile of the *KHDC3L*^c.1A>G^ mole as well as the endometrium of patient D, an unrelated androgenetic mole (AnCHM) and corresponding maternal endometrium, and 2 control placentas. After quality control filtering, methylation data for ~ 745,000 CpG sites for each sample were obtained. We also included methylation data from a further 9 control placentas obtained independently on the same platform to generate a robust control placenta dataset. The *KHDC3L*^c.1A>G^ mole clustered with the AnCHM but separately from the control placentas and endometrium when analysed by principal component analysis (PCA; Additional file 3: Figure S3A). Analysis of 20-kb genomic windows showed a reduction of CpG methylation levels in the *KHDC3L*^c.1A>G^ mole compared to the control placenta (Fig. 1a). However, the general pattern of DNA methylation of the *KHDC3L*^c.1A>G^ mole was reminiscent of that of the control placentas (Fig. 1b). Analysis of genomic features showed that the majority of features, including genes, promoters, exons, introns and intergenic regions, had a reduction in mean methylation of between 6.3 and 9.6% in the *KHDC3L*^c.1A>G^ mole compared with the control placenta, whereas no reduction was observed in CpG islands (CGIs; Additional file 3: Figure S3B). We then specifically evaluated methylated at imprinted gDMRs as listed in Additional file 1: Table S1. For the purpose of this and subsequent analyses, maternal gDMRs are defined as DMRs methylated in oocytes but not in sperm and associated with known imprinted genes (32 features); paternal gDMRs as DMRs methylated in sperm but not oocytes and associated with known imprinted genes (1 feature); placenta-specific gDMRs as CGIs methylated in oocytes but not sperm and retaining allelic differential methylation selectively in placenta (15 features [13];); and secondary gDMRs as elements in known imprinted loci that acquire allelic differential methylation after fertilisation (14 features). In contrast to the small decrease in methylation throughout the genome, there was a substantial reduction in methylation of many maternal gDMRs (23% on average), for example, at the *KCNQ1OT1* locus, whereas paternal gDMRs retained normal methylation (e.g. *H19*) or increased methylation in the case of the secondary DMR at *GNAS-NESP* (Fig. 1b–d, Additional file 3: Figure S3B,C). These methylation abnormalities were confirmed by pyrosequencing at three maternal gDMRs, as well as the normal methylation at the *H19* paternal gDMR (Additional file 3: Figure S3D). The reductions in maternal gDMR methylation in the *KHDC3L*^c.1A>G^ mole were very similar to the AnCHM (Fig. 1c, Additional file 3: Figure S3C) and to those described previously for molar tissue arising from a mutation in *NLRP7* [13]. The placenta methylome is characterised by intermediate methylation levels throughout the genome, with the majority of CGIs being unmethylated (12,889 CGIs < 10%) and 4062 CGIs being methylated (> 70%) [27]. In the *KHDC3L*^c.1A>G^ mole, these characteristics are still evident, and although methylated CGIs and non-CGI regions show decreased DNA methylation, the reduction is significantly less than at maternal gDMRs (Fig. 1e, Additional file 3: Figure S3E, Additional file 4: Table S3). It is possible that the slight hypomethylation of these other genomic features reflects differences in cellular composition in the mole compared with the control placenta.

These findings suggest that the *KHDC3L*^c.1A>G^ conceptus is competent to methylate the genome as a whole during the post-implantation remethylation phase, but that any LoM in gDMRs that might have occurred in the oocyte or preimplantation embryo cannot be fully restored. To evaluate this further, we used the methylation EPIC array data to identify regions with similar hypomethylation as observed for gDMRs in the *KHDC3L*^c.1A>G^ mole compared to the control placentas. To do this, we filtered for 20-kb windows with a mole to control placenta methylation ratio of < 0.65. This analysis identified 479 hypomethylated regions (Additional file 5: Table S4), corresponding to 1.9% of all 20-kb windows analysed (25,030). Of the hypomethylated windows, 17 overlapped with 1 of the 47 primary maternal gDMRs that we included in the study (Additional file 5: Table S4) and a further 27 overlapped with placenta-specific gDMRs identified by Hanna et al. [26]. Together, these results suggest that there is a preferential LoM of imprinted gDMRs in the *KHDC3L*^c.1A>G^ mole, with the remainder of the genome able to establish relatively normal levels of methylation.

### Genome-wide DNA methylation deficit in *KHDC3L*^c.1A>G^ oocytes

We next sought to evaluate genome-wide methylation in oocytes from patient D. Following ovarian stimulation, 1 metaphase-I (MI) and 8 metaphase-II (MII) oocytes were obtained (Additional file 3: Figure S4A), of which the MI and 6 MII oocytes were processed by scBS-seq. Initial sequencing indicated that 5 of the MII oocytes yielded acceptable scBS-seq libraries with no evidence of cumulus cell DNA contamination (Additional file 3: Figure S4B,C). These 5 libraries were then sequenced to saturation, obtaining reads covering 3,249,800–4,354,472 CpG sites or 11.3–15.1% of the genomic total per single-cell library and 43.98% CpG coverage after combining datasets (Additional file 6: Table S5). The scBS-seq data were compared with a published deeply sequenced methylome of bulk germinal vesicle (GV)/MI oocytes [30] and 32 single MII oocyte datasets [31], the latter generated by an equivalent single-cell protocol.

**Fig. 2:**
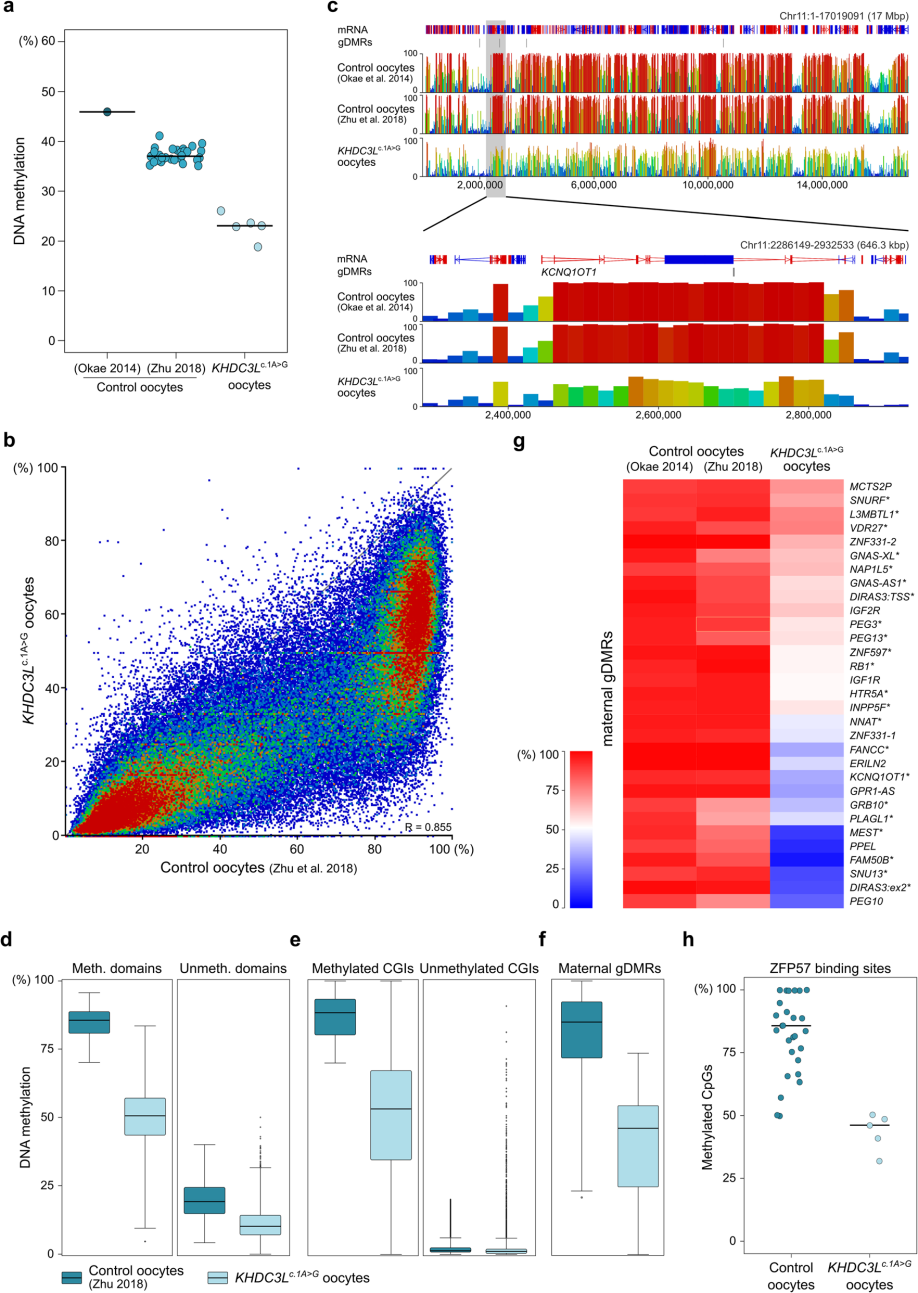
Genome-wide deficit in DNA methylation in *KHDC3L*^c.1A>G^ oocytes. **a** Global CpG methylation as determined by scBS-seq in *KHDC3L*^c.1A>G^ MII oocytes (*n* = 5) compared with PBAT from a bulk population of control GV/MI oocytes (denoted by Okae et al. [30]) and scBS-seq of MII oocytes (*n* = 32, denoted by Zhu et al. [31]). Indicated are mean methylation levels for each single oocyte (dots) and the median of each group. **b** Scatterplot of methylation values of 20-kb windows in grouped *KHDC3L*^c.1A>G^ oocytes (*n* = 5) compared with grouped control oocytes (Zhu et al. [31]; *n* = 32). **c** Seqmonk browser screenshot of genomic methylation in grouped *KHDC3L*^c.1A>G^ oocytes (*n* = 5) compared with bulk (Okae et al. [30]) and grouped (Zhu et al. [31]; *n* = 32) control oocytes. The zoomed-in view shows methylation over the *KCNQ1OT1* imprinted domain. Each vertical bar in both zoomed-out and zoomed-in views is a 20-kb window, height and colour-coded for % methylation. **d**–**f** Box and whisker plots showing methylation in grouped control (Zhu et al. [31]; *n* = 32) and *KHDC3L*^c.1A>G^ oocytes (*n* = 5) of oocyte methylated and unmethylated domains (**d**), methylated and unmethylated CpG islands (CGIs; > 70 and < 20% in control oocytes, respectively) (**e**) and maternal gDMRs (**f**). Boxes represent the interquartile range; lines, the medians; whiskers, the 1.5× the interquartile range; dots beyond the whiskers, outliers. The numbers of features per category are given in Additional file 4: Table S3. **g** Heatmap showing methylation of maternal gDMRs in grouped *KHDC3L*^c.1A>G^ oocytes (*n* = 5) and bulk GV/MI (Okae et al. [30]) or grouped MII (Zhu et al. [31]; *n* = 32) control oocytes. **h** Stripcharts reporting percentage of methylated CpG sites (115) in ZFP57 binding motifs overlapping 32 maternal gDMRs in each single oocyte of control (Zhu et al. [31]; *n* = 32) and *KHDC3L*^c.1A>G^ oocytes (*n* = 5). Dots represent the mean methylation % of all the ZFP57-CpG sites measured in a single oocyte, lines the median of each group. Comparisons in **a** and **d**–**h** are all statistically significant (*p* < 0.0001). Detailed statistical measures are given in Additional file 4: Table S3

The *KHDC3L*^c.1A>G^ oocytes had similarly and substantially reduced global CpG methylation levels compared with control MIIs (median 22.9% versus 37.1%; Fig. 2a, Additional file 3: Figure S5A, Additional file 4: Table S3) and were clearly separated from control oocytes by PCA (Additional file 3: Figure S5B). In comparison, no significant differences were observed for non-CpG methylation (Additional file 3: Figure S5C). Because methylation was similarly affected in the *KHDC3L*^c.1A>G^ oocytes (Fig. 2a, Additional file 3: Figure S5A), most subsequent analysis was done after merging the 5 datasets. Analysis of 20-kb genomic windows showed that the methylation loss in *KHDC3L*^c.1A>G^ oocytes was observed throughout the entire genome, although not every window was affected to the same degree (Fig. 2b). Human oocytes have a distinctive methylation pattern, with coherent domains of high methylation associated with active transcription units and domains of low methylation over non-transcribed regions [30]. This general pattern was preserved in the *KHDC3L*^c.1A>G^ oocytes (Fig. 2c); however, the methylation level of hypermethylated domains was substantially reduced (median 49.1% versus 84.4% in controls; Fig. 2d, Additional file 4: Table S3). We also evaluated methylation of CGIs, which is distinctive in oocytes: 2614 of 22,564 CGIs are normally highly methylated (≥ 70%) in oocytes [30]. Similar to the hypermethylated domains, we found an overall reduction in the methylation of these CGIs (median 53.4% compared with 88.3% in controls; Fig. 2e). When we assessed the maternal gDMRs of imprinted genes, we also found a substantial reduction in methylation of a similar magnitude (median 84.7% in controls, 45.4% in *KHDC3L*^c.1A>G^ oocytes; Fig. 2f, Additional file 4: Table S3). Overall, all genomic features seemed to be losing DNA methylation to a similar extent, although the maternal gDMRs were affected slightly more (Additional file 3: Figure S6A). To test whether maternal gDMRs were actually more affected by LoM than other genomic regions, we identified regions with similar CpG density and methylation levels as maternal gDMRs in control oocytes. Indeed, the average LoM of gDMRs was significantly greater than the comparable control genomic regions (Additional file 3: Figure S6B). Like other genomic elements, imprinted gDMRs were variably affected in *KHDC3L*^c.1A>G^ oocytes, with some loci lacking methylation and others retaining > 50% methylation (Fig. 2f, g). This indicates variable sensitivity to loss of SCMC integrity, but the causes of this variation are unclear. To assess the variation in LoM, we calculated the standard deviation of methylation values of genes and of random, genome-wide 50 CpG windows. The variation in LoM did not appear to be completely random, in that the variation within genes was less than the genome-wide variation (Additional file 3: Figure S6C). However, despite the strong association between methylation establishment and transcription in oocytes [30, 35], the magnitude of LoM was not correlated with transcription level (as inferred from single-cell RNA-seq datasets from healthy oocytes [32]; Additional file 3: Figure S6D). We also assessed the DNA methylation levels in repetitive regions of the genome and found small, but significant, methylation losses in endogenous retroviral elements (ERVs), L1 long-interspersed nuclear elements (LINEs, L1) and short-interspersed nuclear elements (SINEs), whereas no significant changes were observed in long-terminal repeats (LTRs; Additional file 3: Figure S6E).

Because the scBS-seq data do not provide full genome coverage, we did not expect to be able to evaluate methylation of all gDMRs individually in all oocytes. Nevertheless, after setting a coverage threshold of ≥ 10 CpGs/gDMR, we were able to call methylation in at least 2 oocytes for 42 gDMRs (26–33 gDMRs per oocyte). This revealed that many gDMRs exhibited similar LoM (e.g. *ZC3H12C*, *KCNQ1OT1*) in all informative oocytes, while some of those with partial LoM when analysed in the pooled data (Fig. 2g) displayed variation in methylation loss between oocytes (e.g. *PEG3*, *PEG10*; Additional file 3: Figure S7A). This analysis also indicated that some oocytes exhibited greater gDMR LoM overall than others (e.g. MII-6; Additional file 3: Figure S7A,B). Finally, we assessed methylation at binding site motifs within maternal gDMRs for the zinc-finger protein ZFP57 [33, 34], which selectively binds a methylated recognition sequence and is one of the factors required for post-fertilisation maintenance of gDMR methylation [36, 37]. ZFP57-binding motifs also exhibited a reduced rate of methylation in *KHDC3L*^c.1A>G^ oocytes, of a similar magnitude to the loss at gDMRs (Fig. 2h).

### Genome-wide DNA methylation analysis of a *KHDC3L*^c.1A>G^ blastocyst

**Fig. 3:**
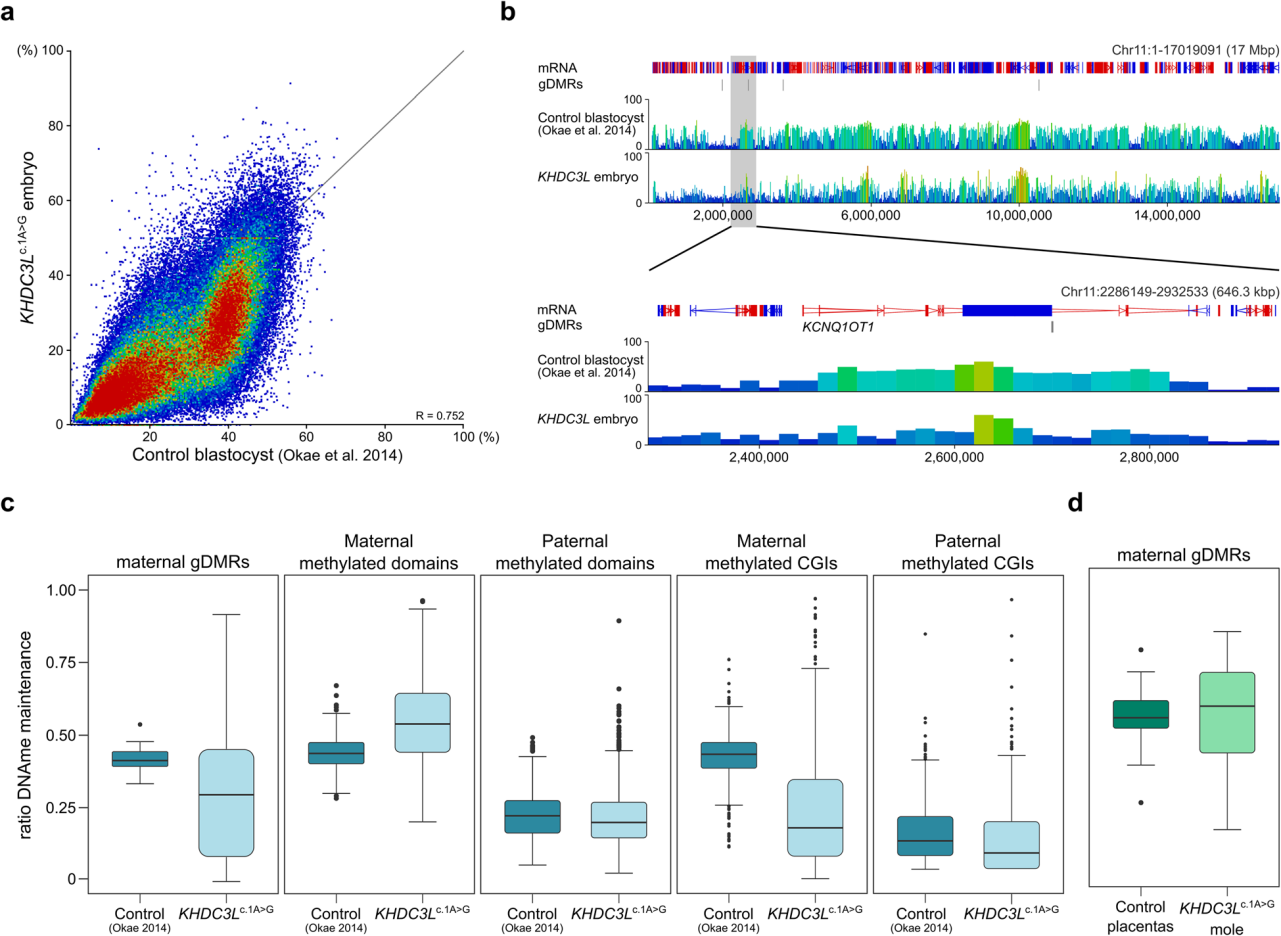
Methylation abnormalities in a *KHDC3L*^c.1A>G^ embryo. **a** Scatterplot comparing methylation determined by PBAT in a single whole *KHDC3L*^c.1A>G^ preimplantation embryo with PBAT of a bulk population of control blastocysts (Okae et al. [30]). Each point is a 20-kb window. **b** Screenshot of methylation in the *KCNQ1OT1* imprinted domain in control blastocysts (Okae et al. [30]) and the *KHDC3L*^c.1A>G^ embryo. Each vertical bar is a 20-kb window, height and colour-coded for % methylation. **c** Box and whisker plots comparing ratio of methylation maintenance in control blastocysts and the *KHDC3L*^c.1A>G^ embryo at maternal gDMRs retaining ≥ 20% DNA methylation in the oocyte (*n* = 38), maternal-methylated domains (*n* = 285), paternal-methylated domains (*n* = 2471), maternal-methylated CGIs (*n* = 735) and paternal-methylated CGIs (*n* = 299). **d** Box and whisker plots comparing methylation maintenance ratio in control blastocysts and the *KHDC3L*^c.1A>G^ mole at maternal and placenta-specific gDMRs (*n* = 40) retaining ≥ 20% DNA methylation in *KHDC3L*^c.1A>G^ oocytes. In **c** and **d**, boxes represent the interquartile range; lines, the medians; whiskers, the 1.5× the interquartile range; and dots beyond the whiskers, outliers. Comparisons in **c** and **d** are all statistically significant (*p* = 0.0125 for maternal gDMRs in **c** and *p* < 0.0001 for all others). Detailed statistical measures are given in Additional file 4: Table S3

The remaining 2 MII oocytes were subject to intra-cytoplasmic sperm injection from which 1 embryo developed in vitro until the late morula/early blastocyst stage at day 6 (Additional file 3: Figure S8A) when it was collected for methylation analysis. Despite being developmentally less advanced than normal blastocysts, the *KHDC3L*^c.1A>G^ embryo had reduced methylation in comparison with control blastocysts [30] (median methylation of 20-kb windows 19.4 versus 28.3%; Fig. 3a, b) and did not cluster with any embryonic stage by PCA (Additional file 3: Figure S8B), suggesting an anomalous methylation pattern. Maternal gDMRs showed variable LoM, as they did in oocytes. (Additional file 3: Figure S8C). A residual, gene body methylation pattern inherited from oocytes is apparent in control blastocysts, and this pattern appears attenuated in the *KHDC3L*^c.1A>G^ embryo (Fig. 3b), as it does in *KHDC3L*^c.1A>G^ oocytes. We inferred the fidelity of methylation maintenance at those gDMRs that had ≥ 20% residual methylation in *KHDC3L*^c.1A>G^ oocytes by calculating the embryo to oocyte methylation ratio, assuming that perfect maintenance would be reflected as a ratio of 0.5. For maternal gDMRs, the actual figure was 0.44 in control blastocysts, but only 0.32 in the *KHDC3L*^c.1A>G^ embryo (median of 38 gDMRs; Fig. 3c, Additional file 4: Table S3). CGIs exclusively methylated in oocytes (maternal methylated CGIs) showed a similar deficit, whereas other sequence features methylated in oocytes (maternal methylated domains) or methylated in sperm (paternal methylated domains and CGIs) were less affected (Fig. 3c). The further reduction in gDMR and maternally methylated CGI methylation in the *KHDC3L*^c.1A>G^ embryo could indicate a role for the SCMC also in methylation maintenance mechanisms. However, when we evaluated methylation at gDMRs in the *KHDC3L*^c.1A>G^ mole, there tended to be a maintenance of the residual levels of methylation from the oocyte (Fig. 3d).

## Discussion

Here, for the first time, we show that an SCMC defect causing BiCHM results in a genome-wide methylation deficit in oocytes. This demonstrates an important application of single-cell bisulphite sequencing in obtaining high-resolution DNA methylation data from very rare material. With the genome-wide coverage obtained, we were able to answer key questions relating to the origins of methylation defects in BiCHM that could not previously be addressed. Importantly, we identified that maternal gDMRs were affected to a similar magnitude as other sequence features that become methylated de novo in oocytes, demonstrating that there is no specificity of the primary defect towards imprinted loci. A primary effect in female gametes is consistent with earlier reports that methylation at the *H19* paternal gDMR is unaffected in BiCHM [12]. That BiCHM tissue in comparison has a generally preserved methylation status, apart from imprinted gDMRs, indicates that these conceptuses are competent for de novo methylation of the genome after implantation, but that defective methylation at gDMRs arising in oocytes cannot be rescued. We also observed a further reduction in gDMR methylation in the single *KHDC3L*^c.1A>G^ preimplantation embryo we were able to assess, which could indicate a role for the integrity of the SCMC also in methylation maintenance mechanisms. Alternatively, the incomplete methylation of CGIs and gDMRs in oocytes, particularly at ZFP57 sites assumed to be critical for methylation maintenance, might allow further erosion of methylation in the preimplantation embryo during the phase of genome-wide methylation reprogramming. We should note, however, that we were only able to assess a single *KHDC3L*^c.1A>G^ preimplantation embryo, which might not be competent for implantation and further development, and may therefore manifest more severe methylation defects than an embryo from which molar tissue could arise.

How could a defect in the SCMC—or in the protein components—impair DNA methylation establishment in the oocyte? The mechanisms of de novo methylation in oocytes are best understood in the mouse, benefitting from genetic manipulations [18]. De novo methylation takes place on a genome largely demethylated after specification of primordial germ cells, in the latter stages of oocyte growth (secondary to antral follicle stage), and culminates in a distinctive methylation landscape with methylation preferentially over expressed gene bodies [21, 30, 35, 38]. Imprinted gDMR methylation is part of this generalised transcription-dependent mechanism [35, 39, 40]. Successful methylation establishment involves the interplay of several nuclear processes. In mice, the required de novo methyltransferase proteins DNMT3A and DNMT3L become abundant in oocytes concomitant with the onset of methylation [41]. Genomic recruitment of DNMT3A/DNMT3L is assumed to depend upon an appropriate chromatin state. DNA methylation coincides with domains of enrichment of histone 3 lysine 36 trimethylation (H3K36me3) over expressed genes, deposited by the unique H3K36me3 methyltransferase SETD2 [42]. Conversely, the histone mark H3K4me3 conventionally enriched at active promoters is antagonistic to DNMT3A/3L recruitment and activity [43, 44], and removal of H3K4 methylation at gDMRs requires transcription-coupled nucleosome remodelling and/or activity of H3K4 demethylases such as KDM1B [35, 45, 46]. The normal methylation pattern also depends upon the exclusion of DNMT1 and its auxiliary protein UHRF1 from the nucleus, which otherwise leads to methylation of intergenic regions: this nuclear exclusion depends on the protein STELLA/PGC7 [47]. How could the global effect on methylation we observe in *KHDC3L*^c.1A>G^ oocytes be explained? Considering the major role of transcription in specifying methylation in oocytes, a global problem in transcription could lead to a generalised deficit in methylation; however, this seems unlikely, as a major effect on transcription sufficient to attenuate methylation to the magnitude observed would likely be incompatible with full development and maturation of the oocyte. RNA-seq analysis of *KHDC3L*^c.1A>G^ oocytes would be required to determine whether transcription defects could account for the variable methylation loss across maternal gDMRs and other genomic features. Gross reductions in the abundance or nuclear localisation of some of the key players above, such as DNMT3A or SETD2, could also cause the effects observed. However, loss of SETD2 in mouse oocytes, in addition to abrogating gene body methylation, leads to substantial methylation gain in intergenic regions [42], which we do not observe in the *KHDC3L*^c.1A>G^ oocytes. The involvement of STELLA in sequestering DNMT1/UHFR1 from the nucleus [47] demonstrates the importance of regulated subcellular localisation of selected epigenetic factors in the oocyte. Conceivably, therefore, normal nuclear localisation of DNMT3A/3L—or other associated factors—in growing oocytes could depend upon an intact SCMC. However, mouse oocytes genetically ablated for the SCMC component NLRP2 are reported to have normal nuclear staining of DNMT3A [17], but they are also unlikely to have such pervasive methylation defects. Moreover, neither *Dnmt3a* or *Dnmt3L* is haploinsufficient in mouse oocytes, indicating that their nuclear availability would need to be reduced to below 50% of normal levels to substantially affect de novo methylation [48, 49]. One mouse gene knockout that phenocopies the global methylation defect of *KHDC3L*^c.1A>G^ oocytes is the oocyte-specific ablation of the histone H3 chaperone HIRA [50]. HIRA is responsible for continuous replacement of H3/H4 during oogenesis, particularly associated with transcription. In addition to grossly diminished de novo methylation, *Hira*-deficient oocytes are severely compromised in ovulation, developmental competence, and chromosome compaction and segregation [50], evidence of the importance of chromatin regulation during oogenesis. It is also important to bear in mind the potential differences between humans and mice in relation to de novo methylation mechanisms in oocytes [18]. Although the striking gene body methylation pattern is conserved, a recent study found differences in the histone regulatory landscape in human compared to mouse oocytes [51], and DNMT3L, an essential co-factor for DNMT3A in the mouse oocyte [48, 52, 53], is not expressed in human oocytes [30, 54]. Furthermore, because KHDC3L, as well as other SCMC members such as NLRP7, do not have mouse orthologues, studying the functional mechanisms by which the SCMC regulates de novo DNA methylation remains challenging.

It was notable that the maternal gDMRs in the *KHDC3L*^c.1A>G^ oocytes, preimplantation embryo and mole exhibited a range of LoM; in addition, there was evidence that individual *KHDC3L*^c.1A>G^ oocytes varied in the severity of gDMR LoM. In comparison, molar tissue from women of the first reported *KHDC3L* pedigree had complete LoM at the few gDMRs evaluated [12]. At present, we do not know the reason for this variation in methylation loss. Similar to our patient D, the mutation in the original pedigree was identified in the initiation codon of *KHDC3L*, but at the + 3 position [4, 12]. In transfections, Parry and co-workers demonstrated the use of the next available in-frame ATG codon (at position 14) for translation of an amino-terminally truncated protein from this mutation [4]. If some residual, truncated protein were translated in vivo, or if other SCMC proteins partially compensate for the loss of KHDC3L, this could account for the partial LoM we observe. Alternatively, if KHDC3L deficiency results in a structural defect in the SCMC that impairs the normal localisation or activity of factors essential for methylation, it might manifest at a time in oocyte growth when de novo methylation is already in progress, such that it attenuates rather than completely prevents methylation. Consistent with this possibility, studies in mice indicate there is asynchrony in the acquisition of methylation of gDMRs and CGIs during oocyte growth [55–57], and recent single-cell analysis of growing and fully-grown oocytes shows some heterogeneity amongst oocytes in methylation of maternal gDMRs [58]. This has some similarity with the variation in gDMR methylation we observe in *KHDC3L*^*c.1A>G*^ oocytes, again consistent with the notion that the progression of de novo methylation during oocyte growth is impaired. Future analysis of the de novo methylation process during normal human oogenesis, as well as knowledge of the timing of elaboration of the SCMC, may help resolve this issue.

## Conclusions

In conclusion, we show that a mutation causing BiCHM results in a substantial and generalised reduction in methylation in oocytes, together with impaired maintenance of gDMR methylation during preimplantation development, and persistence of methylation defects preferentially at imprinted loci post-implantation. The discovery of a primary oocyte defect will now focus attention on how the mechanism of methylation establishment is globally impaired by defects in the SCMC. It also has important implications for possible therapeutic interventions in patient oocytes; these would be very challenging and, if to be considered, would have to aim to restore de novo methylation during oocyte growth, perhaps by injection of *KHDC3L* cRNA into oocytes growing in in vitro follicle culture systems [59–61].

## Supplementary information

**Additional file 1 **: **Table S1:** List of gDMR coordinates; list of ZFP57 binding sites in gDMRs; .xls, 25 KB.

**Additional file 2 **: **Table S2:** Pyrosequencing primer sequences; .xls, 12 KB.

**Additional file 3: Figure S1.** Characterization of Patient D: A. Sequence of *KHDC3L* mutation c.1A>G in patient; B. Extended pedigree. **Figure S2.** Characterization of KHDC3L^c.1A>G^ molar tissue: A. Microsatellite analysis; B. Sequence of *KHDC3L* mutation c.1A>G in molar tissue. **Figure S3.** DNA methylation analysis of *KHDC3L*^*c.1A>G*^ mole: A. Principal component analysis of EPIC array data; B. Barchart of DNA methylation changes of genomic features; C. Heatmap of methylation at imprinted DMRs; D. Pyrosequencing validation of methylation changes at imprinted DMRs; E. Barchart of methylation changes at gDMRs and CpG islands. **Figure S4.** Characterization of *KHDC3L*^*c.1A>G*^ oocytes: A. Photomicrograph of oocytes; B. Principal component analysis of low-depth scBS-seq datasets; C. Genome browser screenshot of low-depth scBS-seq datasets. **Figure S5.** Single-cell DNA methylation analysis of *KHDC3L*^*c.1A>G*^ oocytes: A. Genome browser screenshot of deeply-sequenced scBS-seq datasets; B. Principal component analysis of low-depth scBS-seq datasets; C. Stripchard of non-CpG methylation levels in *KHDC3L*^*c.1A>G*^ oocytes. **Figure S6.** Variability of DNA methylation loss inKHDC3L^*c.1A>G*^ oocytes. A. Barchart of DNA methylation changes at genomic features; B. Box:whisker plot of DNA methylation loss at gDMRs compared with matched non-gDMR features; C. Beanplot of DNA methylation loss over gene bodies; D. Scatterplot of DNA methylation changes correlated with transcript levels; E. Stripchart of DNA methylation changes at repetitive elements. **Figure S7.** Single-cell variation of DNA methylation of imprinted gDMRs in *KHDC3L*^*c.1A>G*^ oocytes. A: Heatmap of methylation of gDMRs and B: Stripchart of methylation of gDMRs in individual oocytes. **Figure S8.** DNA methylation analysis of *KHDC3L*^*c.1A>G*^ embryo. A. Photomicrograph of preimplantation embryo; B. Principal component analysis of methylation of *KHDC3L*^c.1A>G^ embryo and published datasets; C. Heatmap of gDMR methylation in *KHDC3L*^c.1A>G^ embryo. (.word 17.25MB.)

**Additional file 4 : Table S3.** Summary of statistical analysis in Figures and Supplementary Figures; .xls, 24 KB.

**Additional file 5 : Table S4.** Hypomethylated regions in *KHDC3L*^c.1A>G^ mole; .xls, 56 KB

**Additional file 6 : Table S5.** DNA methylation sequencing summary; .xls, 10 KB.

## Data Availability

Sequence and microarray data that support the findings of this study have been deposited under accession codes GSE121056, GSE138864 and GSE122872 in the Gene Expression Omnibus database available at https://www.ncbi.nlm.nih.gov/geo/. Publically available datasets were sourced from DDBJ (https://www.ddbj.nig.ac.jp/index-e.html) and GEO databases: accession DRP002710 [30], GSE81233 [31], GSE17312 (GSM1186665) [27] and GSE44183 [32].

## Abbreviations

AnCHM: Androgenetic complete hydatidiform mole
BiCHM: Biparental complete hydatidiform mole
CGIs: CpG islands
gDMRs: Germline differentially methylated regions
GV: Germinal vesicle
LoM: Loss of methylation
MI: Metaphase I
MII: Metaphase II
MLID: Multi-locus imprinting disorder
PBAT: Post-bisulphite adaptor tagging
PCA: Principal component analysis
scBS-seq: Single-cell bisulphite sequencing
SCMC: Subcortical maternal complex
WGBS: Whole-genome bisulphite sequencing
