# Supplementary material for: A KHDC3L mutation resulting in recurrent hydatidiform mole causes genome-wide DNA methylation loss in oocytes and persistent imprinting defects post-fertilisation
Source: Genome Med. 2019 Dec 17;11:84. doi: 10.1186/s13073-019-0694-y (PMC6918611; doi:10.1186/s13073-019-0694-y)
Supplement: Supplementary file 3 — Additional file 3: Figure S1. Characterization of Patient D: A. Sequence of KHDC3L mutation c.1A>G in patient; B. Extended pedigree. Figure S2. Characterization of KHDC3Lc.1A>G molar tissue: A. Microsatellite analysis; B. Sequence of KHDC3L mutation c.1A>G in molar tissue. Figure S3. DNA methylation analysis of KHDC3Lc.1A>G mole: A. Principal component analysis of EPIC array data; B. Barchart of DNA methylation changes of genomic features; C. Heatmap of methylation at imprinted DMRs; D. Pyrosequencing validation of methylation changes at imprinted DMRs; E. Barchart of methylation changes at gDMRs and CpG islands. Figure S4. Characterization of KHDC3Lc.1A>G oocytes: A. Photomicrograph of oocytes; B. Principal component analysis of low-depth scBS-seq datasets; C. Genome browser screenshot of low-depth scBS-seq datasets. Figure S5. Single-cell DNA methylation analysis of KHDC3Lc.1A>G oocytes: A. Genome browser screenshot of deeply-sequenced scBS-seq datasets; B. Principal component analysis of low-depth scBS-seq datasets; C. Stripchard of non-CpG methylation levels in KHDC3Lc.1A>G oocytes. Figure S6. Variability of DNA methylation loss inKHDC3Lc.1A>G oocytes. A. Barchart of DNA methylation changes at genomic features; B. Box:whisker plot of DNA methylation loss at gDMRs compared with matched non-gDMR features; C. Beanplot of DNA methylation loss over gene bodies; D. Scatterplot of DNA methylation changes correlated with transcript levels; E. Stripchart of DNA methylation changes at repetitive elements. Figure S7. Single-cell variation of DNA methylation of imprinted gDMRs in KHDC3Lc.1A>G oocytes. A: Heatmap of methylation of gDMRs and B: Stripchart of methylation of gDMRs in individual oocytes. Figure S8. DNA methylation analysis of KHDC3Lc.1A>G embryo. A. Photomicrograph of preimplantation embryo; B. Principal component analysis of methylation of KHDC3Lc.1A>G embryo and published datasets; C. Heatmap of gDMR methylation in KHDC3Lc.1A>G embryo. (.word 17.25MB.) [file 13073_2019_694_MOESM3_ESM.docx]

**A *KHDC3L* mutation resulting in recurrent hydatidiform mole causes genome-wide DNA methylation loss in oocytes and persistent imprinting defects post-fertilisation**

Hannah Demond, Zahra Anvar, Bahia Namavar Jahromi, Angela Sparago, Ankit Verma, Maryam Davari, Luciano Calzari, Silvia Russo, Mojgan Akbarzadeh Jahromi, David Monk Simon Andrews, Andrea Riccio, Gavin Kelsey

# Additional file 3: Supplementary Figures

# Supplementary Figures


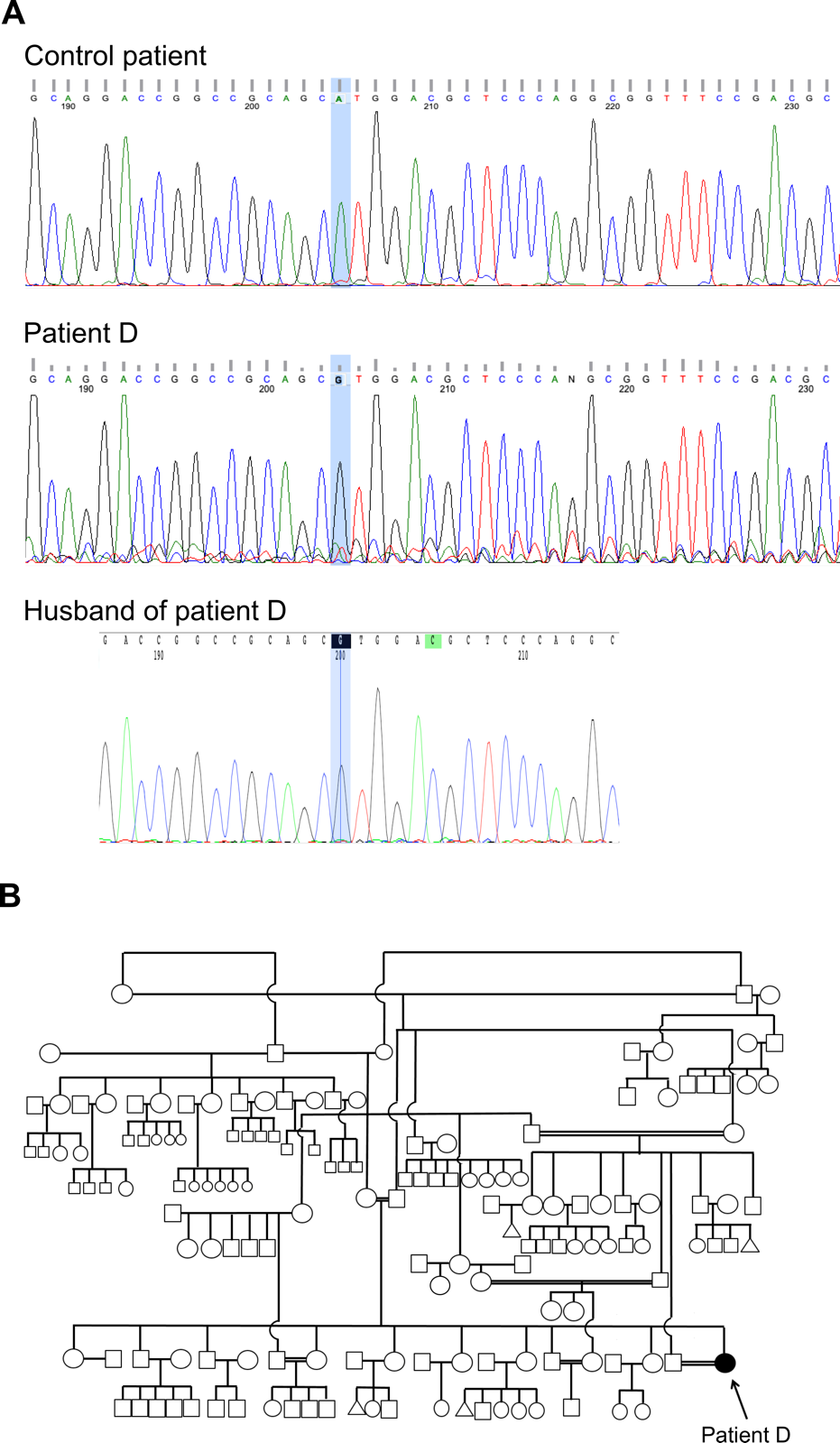


**Supplementary Figure S1: Characterization of Patient D.**

**A,** Sequence trace from Sanger sequencing surrounding the translation initiation codon of the *KHDC3L* gene in Patient D, her husband and a control patient with no mutation in *KHDC3L*. The mutation in the initiation codon is highlighted in blue. **B**, Pedigree of Patient D (indicated in black). Unaffected subjects are indicated in white, females as circles, males as squares, miscarriages as triangles and consanguineous marriages by a double line.

**
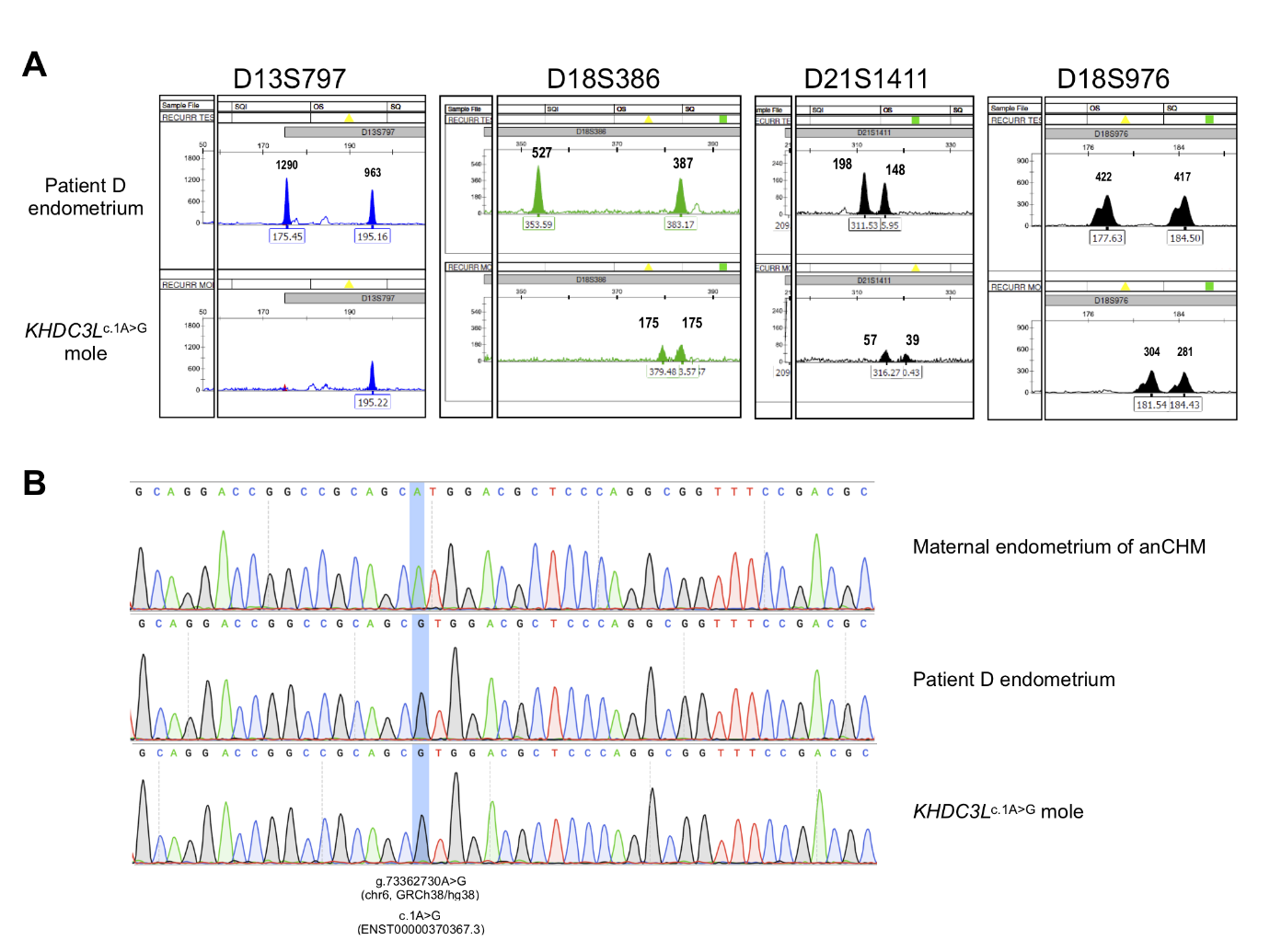
**

**Supplementary Figure S2: Characterization of *KHDC3L*^c.1A>G^ molar tissue﻿.**

**A**, Four microsatellite markers, analysed in endometrium tissue from Patient D and *KHDC3L*^c.1A>G^ molar tissue were found informative. Alleles were separated by capillary electrophoresis and fluorescence measured as peak height (numbers above each allele peak). Only two alleles with balanced peaks were detected in molar tissue, confirming it as biparental CHM. **B**, Sanger sequencing analysis of the region surrounding the ATG initiation codon of *KHDC3L* in the *KHDC3L*^c.1A>G^ mole compared with Patient D endometrium. The mutation is highlighted in blue.

****Supplementary Figure S3: DNA methylation analysis of KHDC3Lc.1A>G mole.

**A**, Principal component analysis (PCA) of CpG methylation determined by the Illumina Human MethylationEPIC BeadChip array comparing molar conceptuses (*KHDC3L*^c.1A>G^ BiCHM and AnCHM) with corresponding endometrium from the same patients and control placentas (n=11) from healthy controls. **B**, Barchart representing absolute loss in DNA methylation of different genomic features in *KHDC3L*^c.1A>G^ molare tissue compared to control placenta (n=11), as determined from Illumina Human MethylationEPIC BeadChip array data. Error bars represent SEM. **C**, Heatmap representing the DNA methylation levels of placenta-specific gDMRs and secondary DMRs in the *KHDC3L*^c.1A>G^ mole, an AnCHM and control placentas (grouped data from 11 placentas). **D**, DNA methylation levels determined by pyrosequencing of three maternal gDMRs (*KCNQ1OT1*, *PLAGL1* and *MEST*) and paternal gDMR *H19* in control placentas (n=2), Patient D endometrium and *KHDC3L*^c.1A>G^ mole. For each gDMR, 5-12 CpGs were tested and the distribution of their % of methylation is represented in the boxplot. Data are a mean of at least two independent PCRs and pyrosequencing experiments. P-values were calculated by two-tailed Student’s T-test (*** = P ≤ 0.001; **** = P ≤ 0.0001). **E,** Mean methylation difference of *KHDC3L*^c.1A>G^ mole compared to control placenta for maternal gDMRs, methylated CGIs, unmethylated CGIs and non-CGI regions. Error bars indicate confidence intervals and P-values were calculated by Brown-Forsythe and Welsh ANOVA followed by Dunnett's T3 multiple comparisons test. Detailed statistical measures are given in **Additional file 4:** **Table S3**.

**Supplementary Figure S4: Characterization of *KHDC3L*^c.1A>G^ oocytes.**

**A**, Oocytes obtained after ovum pick-up from Patient D. **B**, **C**, Initial shallow sequencing (MiSeq) for quality control purposes of scBS-seq libraries from 6 MII and 1 MI *KHDC3L*^c.1A>G^ oocytes. **B**, PCA, based on 100kb overlapping windows (10kb step-size) normally unmethylated (<30%) in oocytes, comparing *KHDC3L*^c.1A>G^ oocytes (n=7) with control single-cell oocyte (n=25) and single sperm (n=32) scBS-seq datasets (1), shows that two *KHDC3L*^c.1A>G^ oocytes (MII-4 and MI-7) cluster separately, close to sperm, which has a somatic-like methylation pattern. **C**, Representative genomic region indicates high DNA methylation for *KHDC3L*^c.1A>G^ oocytes MII-4 and MI-7 in regions that are unmethylated in grouped control oocytes, consistent with somatic cell contamination. These two oocytes were excluded from further analysis. Each vertical bar represents the methylation level of a 100kb window, with the mean methylation % of CpGs in that window represented by the height and colour-coding according to the scale below. Individual CpG calls are indicated in blue (unmethylated) and red (methylated) for each sample, to explain the gaps in coverage due to shallow sequencing. The track on the top are PBAT from a bulk population of control bulk GV/MI oocytes (denoted Okae 2014;(2)) and the grouped scBS-seq of MII oocytes (n=32, denoted Zhu 2018; (1)).

**Supplementary Figure S5: Single-cell DNA methylation analysis of *KHDC3L*^c.1A>G^ oocytes.**

**A**, Representative browser screenshot of scBS-seq profiles of 32 control MII oocytes (1) and 5 *KHDC3L*^c.1A>G^ oocytes, both compared with bulk PBAT data from GV/MI oocytes (2) (denoted Okae 2014). Each vertical bar represents methylation of a 50kb window, with the mean methylation % of CpGs in that window represented by the height and colour-coding according to the scale below. **B**, PCA plot of 50kb windows methylated in control oocytes (>70%) showing that the *KHDC3L*^c.1A>G^ oocytes separate from control MII oocytes on the major principal component, PC1. **C,** non-CpG methylation levels of control oocytes (1, 2) and *KHDC3L*^c.1A>G^ oocytes for chromosome 19. Each dot represents an oocyte, lines indicate the median. No statistical significant differences were observed between the groups (Wilcoxon signed-rank test). Detailed statistical measures are given in **Additional file 4:** **Table S3**.

**Supplementary Figure S6: Variability of DNA methylation loss in *KHDC3L*^c.1A>G^ oocytes. A,** Barchart indicating absolute DNA methylation loss of different genomic features in grouped *KHDC3L*^c.1A>G^ oocytes (n=5) compared to grouped control oocytes (1) (n=32). **B**, DNA methylation loss (%) in grouped *KHDC3L*^c.1A>G^ oocytes (n=5) of 50 CpG windows overlapping maternal gDMRs and non-gDMR windows with matched CpG density and DNA methylation levels in control oocytes. Boxes represent the interquartile range; lines the medians. **C**, Distribution and mean (black line) of standard deviation of CpG methylation within genes. The red line indicates the overall, genome-wide standard deviation (mean of 50 CpG windows), which is higher than the standard deviation within genes, demonstrating that DNA methylation variation is greater between genes than within genes. **D**, Scatterplot of DNA methylation loss (change in %) in *KHDC3L*^c.1A>G^ oocytes compared with gene expression (log2 FPKM) in control human oocytes (pooled scRNA-seq data from (3)). **E,** DNA methylation levels of different classes of repetitive elements in *KHDC3L*^c.1A>G^ oocytes (grouped, n=5) compared to grouped control oocytes (1) (n=32). Each dot represents DNA methylation levels of a consensus sequence of a repetitive element within a class in the indicated pooled oocyte scBS-seq datasets. Lines indicate medians. P-values of Wilcoxon signed-rank test are shown. Detailed statistical measures are given in **Additional file 4:** **Table S3**.

**Supplementary Figure S7: Single-cell variation of DNA methylation of imprinted gDMRs in *KHDC3L*^c.1A>G^ oocytes.**

Heatmap (**A**) and box:whisker plots (**B**) of DNA methylation at maternal gDMRs of grouped control (n=32) and *KHDC3L*^c.1A>G^ (n=5) oocytes, compared to methylation levels in individual *KHDC3L*^c.1A>G^ oocytes. **A**, gDMRs with insufficient coverage in individual *KHDC3L* ^c.1A>G^ oocytes (<10 informative CpGs) are indicated in grey. In **B**, jitter indicates methylation of individual gDMRs, the boxes the interquartile range, with the horizontal bar median values.


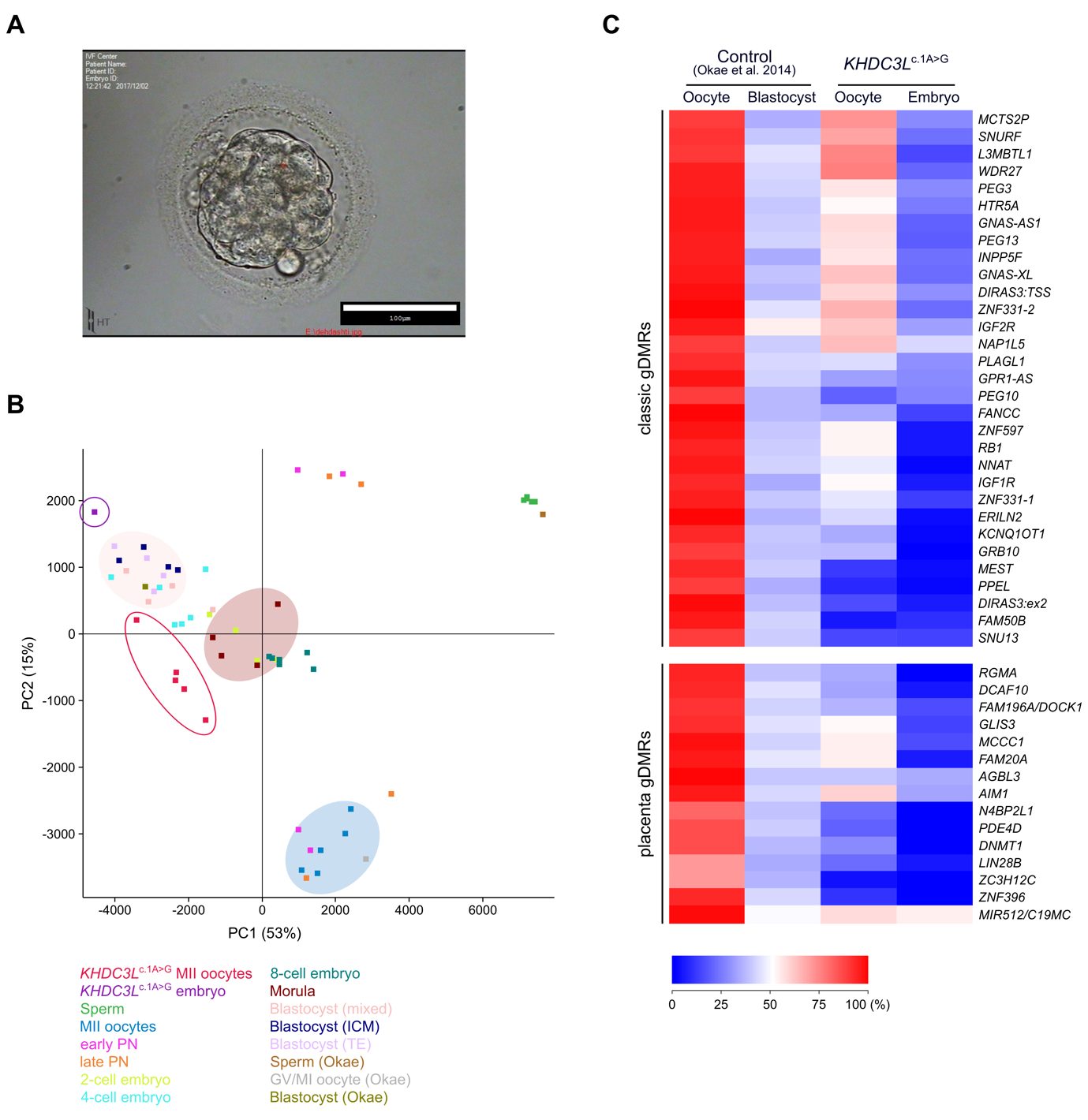


**Supplementary Figure S8: DNA methylation analysis of *KHDC3L*^c.1A>G^ embryo.**

**A**, Embryo produced by ICSI from Patient D oocyte at day 6 of embryo culture. **B**, PCA of methylation levels of 500 CpG windows of single-cell data (from (1)) and bulk data (from (2); indicated Okae in the legend) compared to single-cell oocyte and single-embryo data from Patient D. Patient D’s oocytes and embryo are highlighted with open circles, whereas developmentally comparable control oocyte (blue), morula (brown; comparable in cell number to *KHDC3L*^c.1A>G^ embryo) and blastocyst (light pink; comparable in developmental timing to *KHDC3L*^c.1A>G^ embryo) samples are highlighted by shaded circles. **C**, Heatmap of methylation levels of classic imprinted gDMRs and placenta-specific gDMRs in control oocytes and blastocysts (bulk PBAT data from (2)), and *KHDC3L*^c.1A>G^ oocytes (pooled data, n=5) and embryo.

# References

1. Zhu P, Guo H, Ren Y, Hou Y, Dong J, Li R, et al. Single-cell DNA methylome sequencing of human preimplantation embryos. Nat Genet. 2018;50(1):12-9.

2. Okae H, Chiba H, Hiura H, Hamada H, Sato A, Utsunomiya T, et al. Genome-wide analysis of DNA methylation dynamics during early human development. PLoS Genet. 2014;10(12):e1004868.

3. Xue Z, Huang K, Cai C, Cai L, Jiang CY, Feng Y, et al. Genetic programs in human and mouse early embryos revealed by single-cell RNA sequencing. Nature. 2013;500(7464):593-7.
